# Serum trihalomethanes and cognitive decline: investigating environmental risk factors for neurodegenerative diseases

**DOI:** 10.3389/fpubh.2025.1603138

**Published:** 2025-06-27

**Authors:** Jianping Liu, Sufang Wang, Yuanying Song, Rong Luo, Lijian Han

**Affiliations:** ^1^Department of Neurology, Yancheng Third People's Hospital (The Sixth Affiliated Hospital of Nantong University, The Yancheng School of Clinical Medicine of Nanjing Medical University, The Affiliated Hospital of Jiangsu Vocational College of Medicine), Yancheng, Jiangsu, China; ^2^School of Pharmacy, Nanjing Medical University, Nanjing, Jiangsu, China

**Keywords:** trihalomethanes, cognitive impairment, neurotoxicity, NHANES, environmental exposure

## Abstract

**Backgrounds:**

Trihalomethanes (THMs), byproducts of water chlorination, are pervasive in drinking water supplies and have known systemic toxicity. However, their potential neurotoxic effects, particularly on cognitive function, remain poorly understood. This study investigates the association between serum THM concentrations and cognitive decline, aiming to identify environmental risk factors for neurodegenerative diseases.

**Methods:**

Data were drawn from the National Health and Nutrition Examination Survey (NHANES) 2011–2014 cohort. A final analytic sample of 743 participants aged 60 years or older was analyzed. Serum concentrations of four THM species—chloroform, bromodichloromethane (BDCM), dibromochloromethane (DBCM), and bromoform (TBM)—were measured. Cognitive performance was assessed using CERAD Word Learning and Delayed Recall, animal fluency test (AFT), and digit symbol substitution test (DSST). Cognitive impairment was defined as scores below the 25th percentile. Multivariate logistic regression, restricted cubic splines (RCS), and subgroup interaction analyses were used to explore associations.

**Results:**

Higher serum THM concentrations were significantly associated with increased odds of cognitive impairment. In the fully adjusted model, individuals in the highest quartile of total THMs (TTHMs) had a 2.50-fold higher risk (95% CI: 1.68–3.71) compared to the lowest quartile. RCS analysis revealed a non-linear association between BDCM and cognitive decline, particularly in the AFT. Subgroup analysis indicated that older adults (≥70 years), females, and individuals with hypertension or diabetes were more susceptible to THM-related cognitive impairment.

**Conclusion:**

Elevated serum THM levels are independently associated with cognitive impairment, particularly in vulnerable populations. These findings suggest that THMs may act as environmental neurotoxicants contributing to cognitive decline. Public health efforts to reduce THM exposure could play a role in mitigating the risk of neurodegenerative diseases.

## 1 Introduction

Trihalomethanes (THMs) are a class of chemical compounds formed as unintended byproducts during the chlorination of drinking water (1). These compounds, including chloroform, bromoform (TBM), dibromochloromethane (DBCM), and chlorodibromomethane, have been a subject of increasing concern due to their widespread presence in potable water supplies (2). While much research has focused on the toxicological effects of THMs on liver, kidney, and cardiovascular health, their potential impact on neurological function, particularly cognitive decline, remains underexplored (3, 4). The neurotoxic potential of environmental chemicals, especially those involved in long-term exposure like THMs, could have profound implications for public health, as cognitive dysfunction is linked to numerous age-related conditions such as dementia and Alzheimer's disease (5, 6).

This study utilizes data from the National Health and Nutrition Examination Survey (NHANES) conducted between 2011 and 2014 to investigate the relationship between serum THM concentrations and cognitive health. Cognitive dysfunction, which encompasses a wide range of disorders from mild cognitive impairment to severe dementia, is one of the leading public health concerns globally (7). The increasing incidence of neurodegenerative diseases underscores the urgency of identifying modifiable environmental risk factors (8). While the role of chemical exposures in the development of cognitive decline has been studied in various contexts, research on the specific effects of THMs remains limited and inconclusive (9). Therefore, understanding the potential mechanisms through which THMs may influence brain health is critical.

The pathophysiological mechanisms by which THMs may impair cognitive function are still not fully understood, though several theories exist. One proposed mechanism involves oxidative stress, where THMs induce the production of free radicals that cause cellular damage in brain tissues, particularly neurons (10, 11). Additionally, inflammation and neuroinflammation could play a pivotal role in this process, as prolonged exposure to environmental pollutants is known to activate the immune response in the brain, leading to chronic inflammatory states that affect cognitive function (12). Furthermore, THMs may also disrupt the blood-brain barrier (BBB) or interfere with neurotransmitter systems, exacerbating the risk of cognitive decline (13). Given these potential mechanisms, the neurotoxic effects of THMs could be subtle and cumulative, manifesting over time with increasing exposure (14).

Our findings suggest a significant association between elevated serum THM concentrations and an increased risk of cognitive impairment. These results highlight the potential neurotoxic effects of THMs, positioning them as environmental risk factors for cognitive decline (15). From a public health perspective, this study underscores the importance of reducing exposure to such chemicals, particularly in populations that may be more vulnerable, such as the older person and those living in areas with suboptimal water treatment. While the findings of this study are preliminary, they pave the way for further research to explore the causal relationship between THM exposure and cognitive decline, and to assess the efficacy of public health policies aimed at limiting exposure to these harmful substances.

In conclusion, the implications of THM exposure for cognitive health are far-reaching. As the global population continues to age, understanding the environmental determinants of cognitive decline is essential to preventing and managing neurodegenerative diseases. This research not only contributes to the existing body of knowledge but also calls for urgent action to safeguard public health by minimizing the presence of harmful environmental pollutants such as THMs in drinking water supplies.

## 2 Methods

### 2.1 Study population

**Figure 1:**
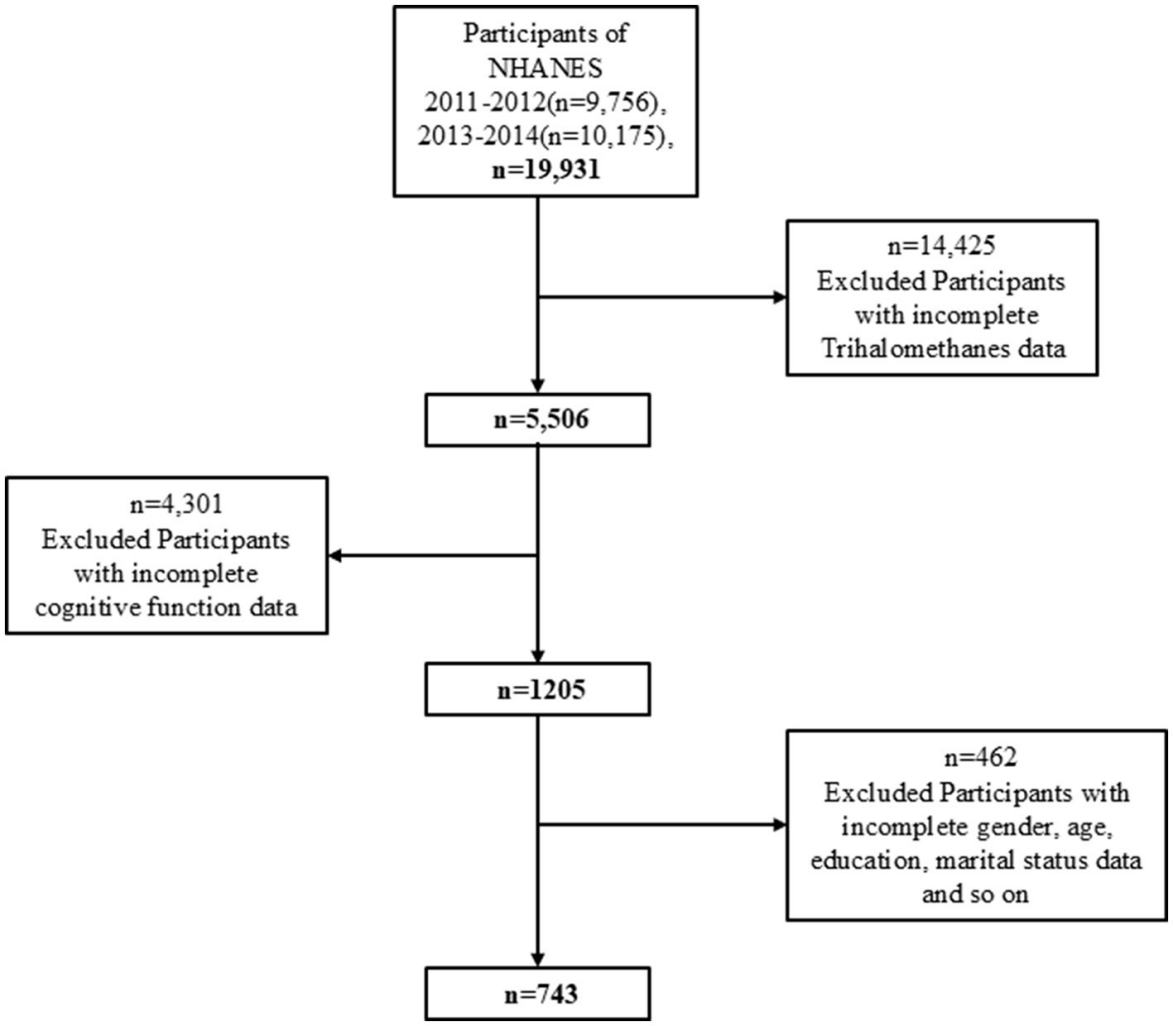
Flowchart of the screening process for the study population.

Data for this study were derived from the National Health and Nutrition Examination Survey (NHANES) 2011–2014 cohort. We limited our analysis to the NHANES 2011–2014 cycles because these are the only cycles in which both serum trihalomethane (THM) concentrations and cognitive function assessments (CERAD-WL, CERAD-DR, AFT, and DSST) were simultaneously collected. Figure 1 illustrates the data selection process used in our study. Initially, 19,931 participants were included, but exclusions were made for individuals with missing serum trihalomethane (THM) data (*n* = 14,425), resulting in a sample of 5,506 participants. Further exclusions for missing cognitive data (*n* = 4,301) left 1,205 participants. After removing individuals with missing covariate data, the final analytic sample consisted of 743 participants.

### 2.2 Exposure assessment

Serum THM concentrations were assessed in peripheral blood samples, as previously described in the literature. THMs measured included chloroform (TCM), bromodichloromethane (BDCM), dibromochloromethane (DBCM), and bromoform (TBM). For analytes with results below the limit of detection (LOD), values were substituted with LOD/√2. Additionally, total THM (TTHMs), chlorinated THMs (Cl-THMs, comprising TCM, BDCM, and DBCM), and brominated THMs (Br-THMs, consisting of BDCM, DBCM, and TBM) concentrations were calculated (16). These measures provided a comprehensive exposure assessment of THMs as potential neurotoxicants.

### 2.3 Outcome assessment: cognitive performance

Cognitive performance was evaluated using several standardized tests included in the NHANES 2011–2014 cycles, specifically the Consortium to Establish a Registry for Alzheimer's Disease Word List Learning (CERAD-WL), CERAD Delayed Recall (CERAD-DR), animal fluency test (AFT), and digit symbol substitution test (DSST). The CERAD test evaluates episodic memory through immediate word list learning and delayed recall. Each participant was asked to learn and recall ten words, and the total score for the CERAD-WL and CERAD-DR tests combined could range from 0 to 40. The 25th percentile threshold was selected based on precedents in population-based cognitive studies using NHANES, particularly when standardized clinical cutoffs are unavailable. This approach allows for the identification of individuals with relatively poor performance within a population distribution. Nonetheless, we acknowledge the limitation and note that future work may benefit from incorporating age- and education-adjusted clinical norms.

The AFT measures categorical verbal fluency by asking participants to name as many animals as possible in 1 min. A score of one point was awarded for each correct animal named. The DSST, a test of cognitive processing speed and sustained attention, requires participants to match symbols to corresponding numbers within 2 min, yielding a total score of 133 (17).

To define cognitive impairment, we used the 25th percentile cutoff of each cognitive test score, as established in prior research. This approach has been widely used to identify lower cognitive performance in population-based studies.

### 2.4 Covariates

Potential confounding variables included sex, age, race, education level, marital status, income-to-poverty ratio (PIR), smoking status, alcohol consumption, hypertension, hyperlipidemia, and diabetes. These covariates were selected based on established associations with both THM exposure and cognitive function in the literature.

### 2.5 Statistical analysis

Descriptive statistics were employed to summarize baseline characteristics. Differences between groups were assessed using *t*-tests for continuous variables and chi-square tests for categorical variables. Chi-square tests were used for categorical variables and ANOVA for continuous variables to assess differences in demographic and health characteristics across quartiles of THM exposure.

The relationship between serum THM levels and cognitive performance was analyzed using multivariate logistic regression in three models:

- Model 1 included only the exposure (THM concentrations) and the outcome (cognitive performance).
- Model 2 adjusted for sex and age.
- Model 3 incorporated all selected covariates (sex, age, race, education, marital status, PIR, smoking, alcohol consumption, hypertension, hyperlipidemia, and diabetes).

To assess potential non-linear associations between THM concentrations and cognitive outcomes, restricted cubic splines (RCS) were used. Subgroup analyses were conducted to explore whether the relationship between THMs and cognitive impairment differed by demographic or health status factors. Interaction terms were also included to examine potential effect modification.

All statistical analyses were conducted using SPSS version 27.0 and R version 4.4.2. Statistical significance was defined as a *P*-value of < 0.05. All statistical analyses incorporated the NHANES sampling weights to ensure national representativeness and account for the complex survey design. To combine the 2011–2012 and 2013–2014 cycles, 4-year weights were calculated by dividing the 2-year weights by two, as recommended by NHANES guidelines (https://wwwn.cdc.gov/nchs/nhanes/tutorials/weighting.aspx).

## 3 Results

### 3.1 Baseline characteristics

The final analytic sample included 743 participants, stratified into quartiles based on total serum THM concentrations (Table 1). The mean age was 69.83 ± 6.71 years, and 55.8% of participants were female. Significant differences were observed in demographic, socioeconomic, and health-related variables across THM quartiles (all *P* < 0.001). For example, the proportion of Non-Hispanic Black individuals was highest in the lowest THM quartile (Q1: 53.3%) and decreased in higher quartiles. Participants in the highest quartile (Q4) tended to have lower educational attainment (only 0.9% had college degrees or above) and a higher prevalence of hypertension (67.3%) and hyperlipidemia (59.1%) compared to lower quartiles. Additionally, smoking and alcohol consumption were more common in higher exposure groups. These differences underscore the necessity of adjusting for multiple covariates in subsequent models.

**Table 1:** Baseline characteristics of the study participants.

**Characteristics**	**Overall**	**Blood total trihalomethanes**				***P*-value**
		**Q1**	**Q2**	**Q3**	**Q4**	
*n*	743	212	197	279	55	
Age, years	69.83 ± 6.71	69.58 ± 6.85	69.67 ± 6.50	70.01 ± 6.62	70.49 ± 7.50	< 0.001
**Gender**, ***n*** **(%)**						< 0.001
Male	329 (44.2%)	100 (13.5%)	88 (11.8%)	128 (17.2%)	13 (1.7%)	
Female	414 (55.8%)	112 (15.1%)	109 (14.7%)	151 (20.3%)	42 (5.7%)	
**Race**, ***n*** **(%)**						< 0.001
Mexican American	56 (7.5%)	9 (1.2%)	15 (2.0%)	31 (4.2%)	1 (0.1%)	
Other Hispanic	62 (8.4%)	29 (3.9%)	19 (2.6%)	12 (1.6%)	2 (0.3%)	
Non-Hispanic Black	399 (53.7%)	113 (15.2%)	96 (12.9%)	164 (22.1%)	26 (3.5%)	
Non-Hispanic White	148 (19.9%)	45 (6.1%)	47 (6.3%)	39 (5.2%)	17 (2.3%)	
Other races	78 (10.5%)	16 (2.2%)	20 (2.7%)	33 (4.4%)	9 (1.2%)	
**Education**, ***n*** **(%)**						< 0.001
< 9th grade	76 (10.2%)	29 (3.9%)	14 (1.9%)	28 (3.7%)	5 (0.7%)	
9–11th grade	87 (11.7%)	28 (3.8%)	22 (3.0%)	29 (3.9%)	8 (1.0%)	
High school graduate	190 (25.6%)	59 (7.9%)	48 (6.5%)	67 (9.0%)	16 (2.2%)	
Some college or AA degree	216 (29.1%)	45 (6.0%)	68 (9.2%)	84 (11.3%)	19 (2.6%)	
College graduate or above	174 (23.4%)	51 (6.9%)	45 (6.1%)	71 (9.5%)	7 (0.9%)	
**Marital status**, ***n*** **(%)**						< 0.001
Married	407 (54.8%)	103 (13.9%)	115 (15.4%)	161 (21.7%)	28 (3.8%)	
Widowed	155 (20.9%)	46 (6.2%)	38 (5.1%)	53 (7.1%)	18 (2.5%)	
Divorced	114 (15.3%)	41 (5.5%)	27 (3.6%)	40 (5.4%)	6 (0.8%)	
Separated	12 (1.6%)	5 (0.7%)	5 (0.7%)	2 (0.2%)	0 (0.0%)	
Never married	41 (5.5%)	12 (1.6%)	7 (0.9%)	19 (2.6%)	3 (0.4%)	
Living with partner	14 (1.9%)	5 (0.7%)	5 (0.7%)	4 (0.5%)	0 (0.0%)	
**PIR**, ***n*** **(%)**						< 0.001
≤ 1	125 (16.8%)	33 (4.4%)	29 (3.9%)	54 (7.3%)	9 (1.2%)	
1–3	334 (44.9%)	108 (14.5%)	76 (10.2%)	123 (16.6%)	27 (3.6%)	
>3	284 (38.3%)	71 (9.6%)	92 (12.4%)	102 (13.7%)	19 (2.6%)	
**Smoke**, ***n*** **(%)**						< 0.001
Yes	336 (45.2%)	105 (14.1%)	86 (11.6%)	129 (17.3%)	16 (2.2%)	
No	407 (54.8%)	107 (14.4%)	111 (14.9%)	150 (20.2%)	39 (5.3%)	
**Alcohol use**, ***n*** **(%)**						< 0.001
Yes	493 (66.4%)	133 (17.9%)	138 (18.6%)	193 (26.0%)	29 (3.9%)	
No	250 (33.6%)	79 (10.6%)	59 (7.9%)	86 (11.6%)	26 (3.5%)	
**Hypertension**, ***n*** **(%)**						< 0.001
Yes	465 (62.6%)	140 (18.9%)	122 (16.4%)	166 (22.3%)	37 (5.0%)	
No	278 (37.4%)	72 (9.7%)	75 (10.1%)	113 (15.2%)	18 (2.4%)	
**Hyperlipidemia**, ***n*** **(%)**						< 0.001
Yes	411 (55.3%)	120 (16.1%)	115 (15.5%)	149 (20.1%)	27 (3.6%)	
No	332 (44.7%)	92 (12.4%)	82 (11.0%)	130 (17.5%)	28 (3.8%)	
**Diabetes**, ***n*** **(%)**						< 0.001
Yes	159 (21.4%)	44 (5.9%)	43 (5.8%)	59 (7.9%)	13 (1.8%)	
Borderline	547 (73.6%)	153 (20.6%)	144 (19.4%)	208 (28.0%)	42 (5.6%)	
No	37 (5.0%)	15 (2.0%)	10 (1.4%)	12 (1.6%)	0 (0.0%)	

### 3.2 Multivariate logistic regression analysis

The results of the multivariate logistic regression analysis examining the association between serum THM concentrations and cognitive impairment are presented in Table 2. The analysis was conducted in three models: Model 1 (unadjusted), Model 2 (adjusted for age, sex, and race), and Model 3 (fully adjusted for all covariates).

**Table 2:** Weighted logistic regression analyses of association between the blood trihalomethane concentrations and cognitive impairment.

***Z*-score**	**Model 1**		**Model 2**		**Model 3**	
	**OR 95% CI**	***P*** **value**	**OR 95% CI**	***P*** **value**	**OR 95% CI**	***P*** **value**
Q1	Ref		Ref		Ref	
Q2	1.01 (0.70, 1.47)	0.949	1.15 (0.78, 1.70)	0.494	1.08 (0.73, 1.62)	0.692
Q3	0.89 (0.61, 1.30)	0.550	1.27 (0.85, 1.90)	0.240	1.17 (0.77, 1.76)	0.464
Q4	1.46 (1.03, 2.07)	0.032	2.86 (1.95, 4.21)	< 0.001	2.50 (1.68, 3.71)	< 0.001
*P* for trend	< 0.001		< 0.001		< 0.001	

Model 1: no covariates were adjusted.

Model 2: age, sex, and race were adjusted.

Model 3: age, sex, race, education level, marital status, BMI, PIR, smoking status, alcohol status, diabetes status, hypertension status, hyperlipidemia status was adjusted.

95% CI, 95% confidence interval.

In Model 1, the odds ratios (ORs) for cognitive impairment in the second (Q2), third (Q3), and fourth (Q4) quartiles of serum THM concentrations relative to the first quartile (reference group) were 1.01 (95% CI: 0.70, 1.47), 0.89 (95% CI: 0.61, 1.30), and 1.46 (95% CI: 1.03, 2.07), respectively. The *P*-value for the trend across quartiles was < 0.001, indicating a significant increasing trend in the odds of cognitive impairment with higher THM concentrations. Although individual comparisons for Q2 and Q3 were not statistically significant, the overall dose–response relationship remains evident, as supported by a significant trend test (*P* for trend < 0.001).

After adjusting for age, sex, and race in Model 2, the OR for the fourth quartile (Q4) increased to 2.86 (95% CI: 1.95, 4.21), and the *P*-value for the trend remained significant (*P* < 0.001). In the fully adjusted Model 3, which included additional covariates (education level, marital status, PIR, smoking status, alcohol use, diabetes, hypertension, and hyperlipidemia), the OR for the fourth quartile of THMs was 2.50 (95% CI: 1.68, 3.71), further supporting a robust association between higher THM concentrations and cognitive impairment.

### 3.3 Restricted cubic spline analysis

**Figure 2:**
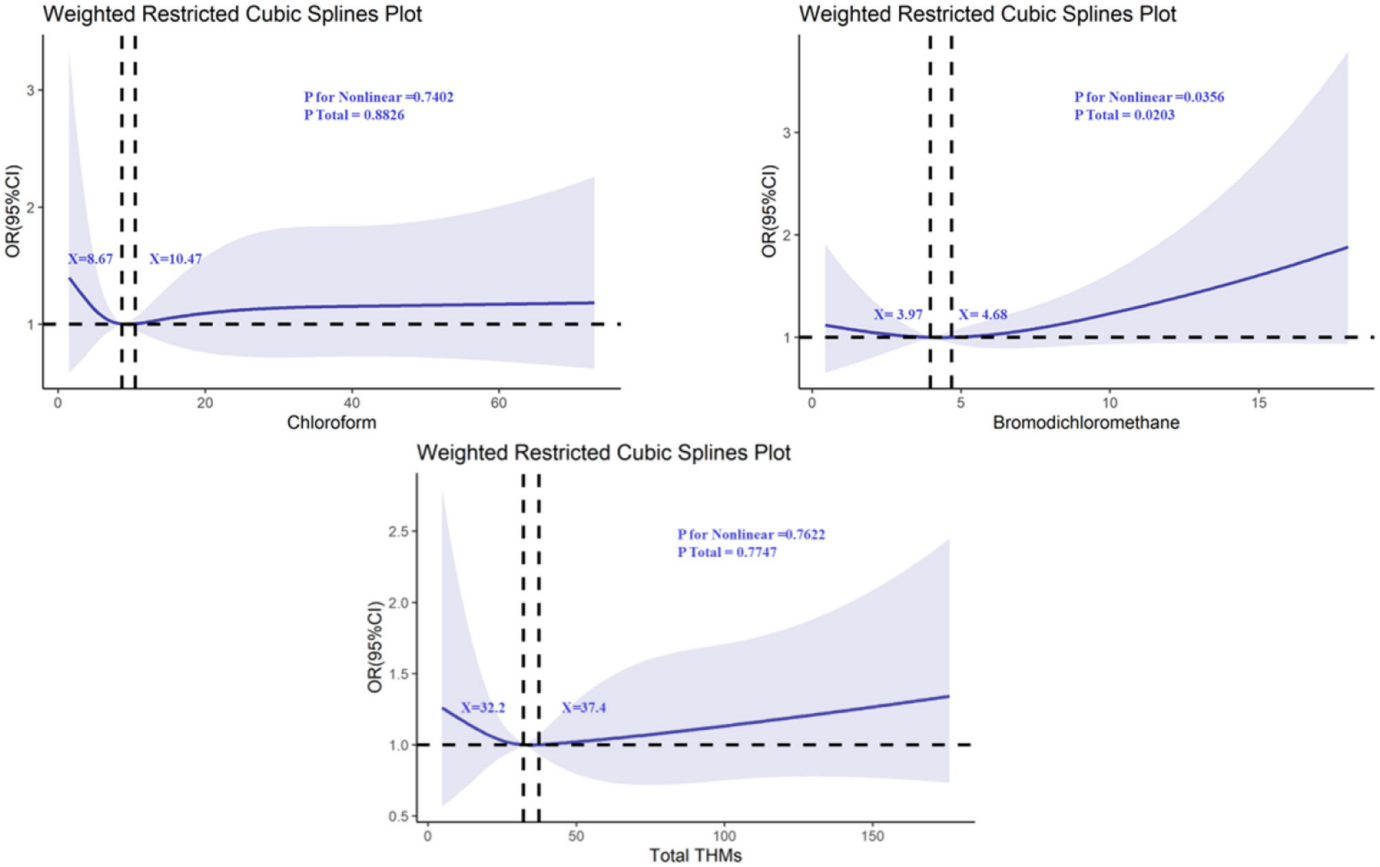
Determination of the association between trihalomethane and animal fluency test by restricted cubic spline (RCS) regression analysis.

The non-linear relationships between individual THMs and cognitive function were evaluated using restricted cubic splines (RCS), focusing on chloroform (TCM), bromodichloromethane (BDCM), dibromochloromethane (DBCM), and bromoform (TBM). The analysis revealed a significant non-linear relationship between BDCM and cognitive performance on the AFT test. Specifically, there were two distinct inflection points at serum BDCM concentrations of 3.97 and 4.68 (Figure 2), with a *P*-value for non-linearity of 0.0356. This suggests that the relationship between BDCM levels and cognitive performance is not linear, and the risk of cognitive impairment may increase more sharply beyond these concentrations. In addition to total THMs, we conducted species-specific analyses for chloroform, bromodichloromethane (BDCM), dibromochloromethane, and bromoform. These analyses revealed that bromoform was associated with the AFT test (Supplementary Table 1).

**Figure 3:**
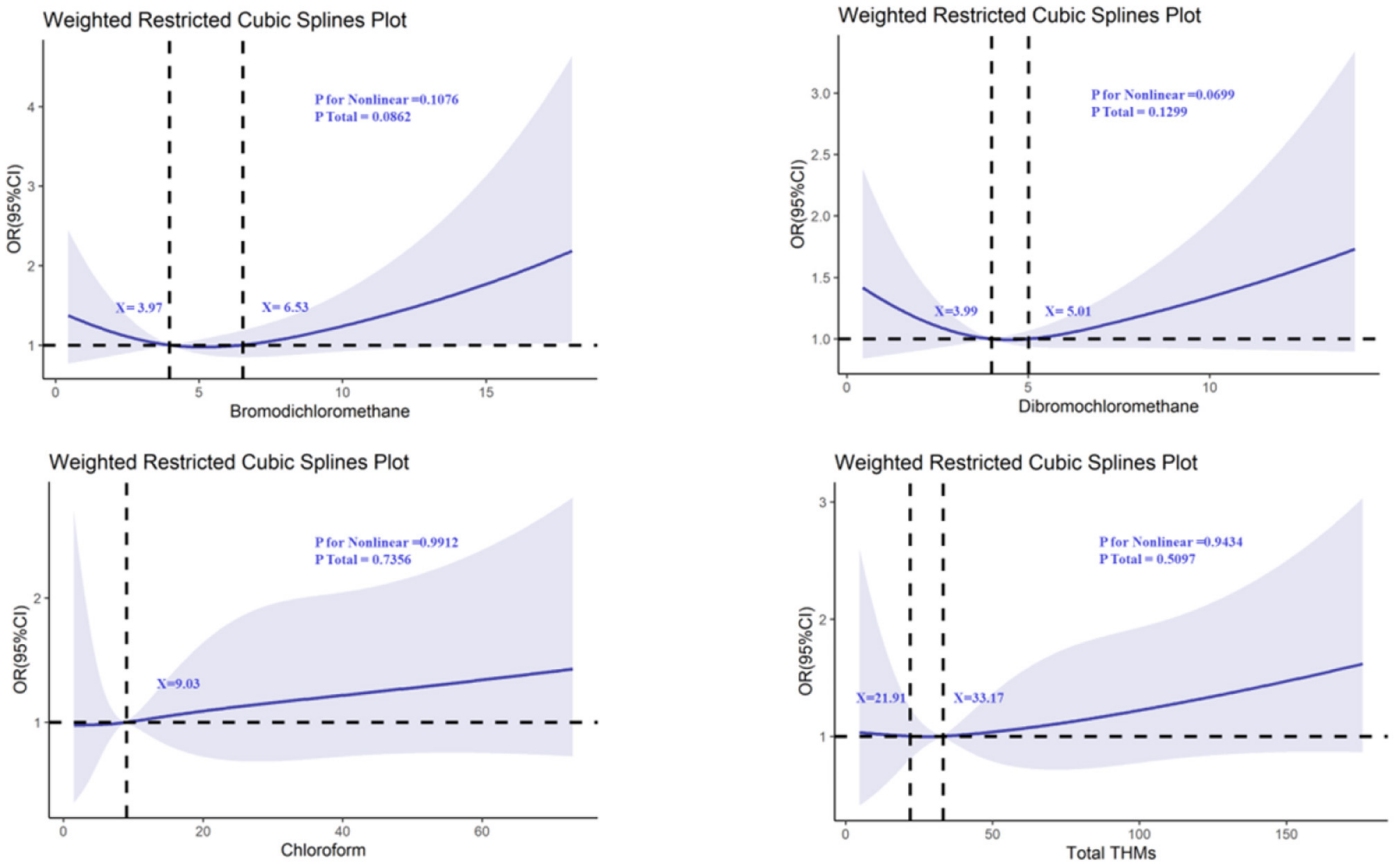
Determination of the association between trihalomethane and Consortium to Establish a Registry for Alzheimer's Disease-Word Learning (CERAD W-L) by restricted cubic spline (RCS) regression analysis.

**Figure 4:**
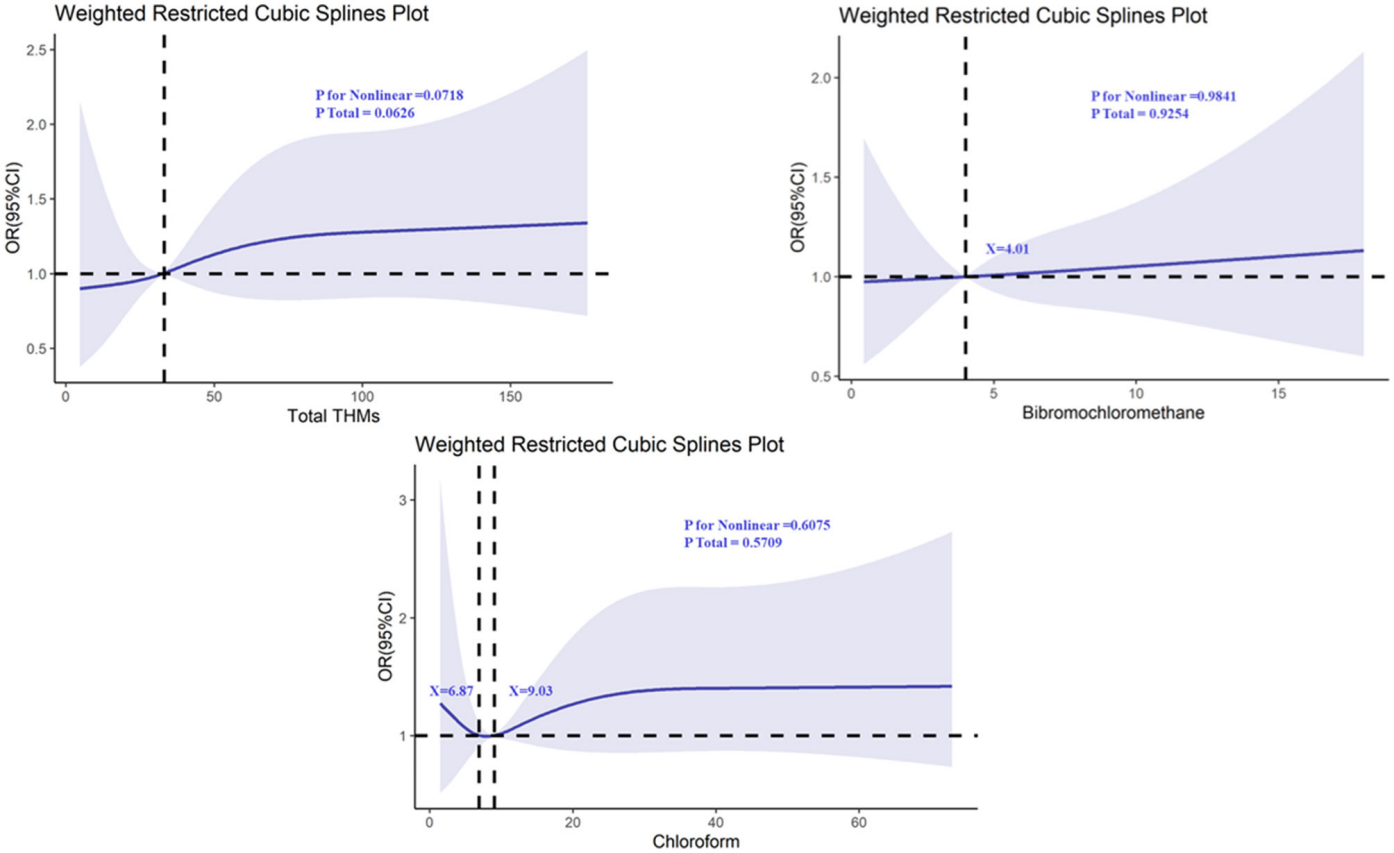
Determination of the association between trihalomethane and digit symbol substitution test (DSST) by restricted cubic spline (RCS) regression analysis.

For the other THMs (TCM, DBCM, and TBM), while the *P*-values for non-linearity were not statistically significant, the RCS plots showed that all of them intersected with an odds ratio of 1, suggesting that there might be thresholds or subtle effects at higher levels of exposure (Figures 3, 4). These findings highlight the need for further investigation into the potential thresholds or nonlinear effects of THMs on cognitive function.

### 3.4 Subgroup and interaction analyses

**Figure 5:**
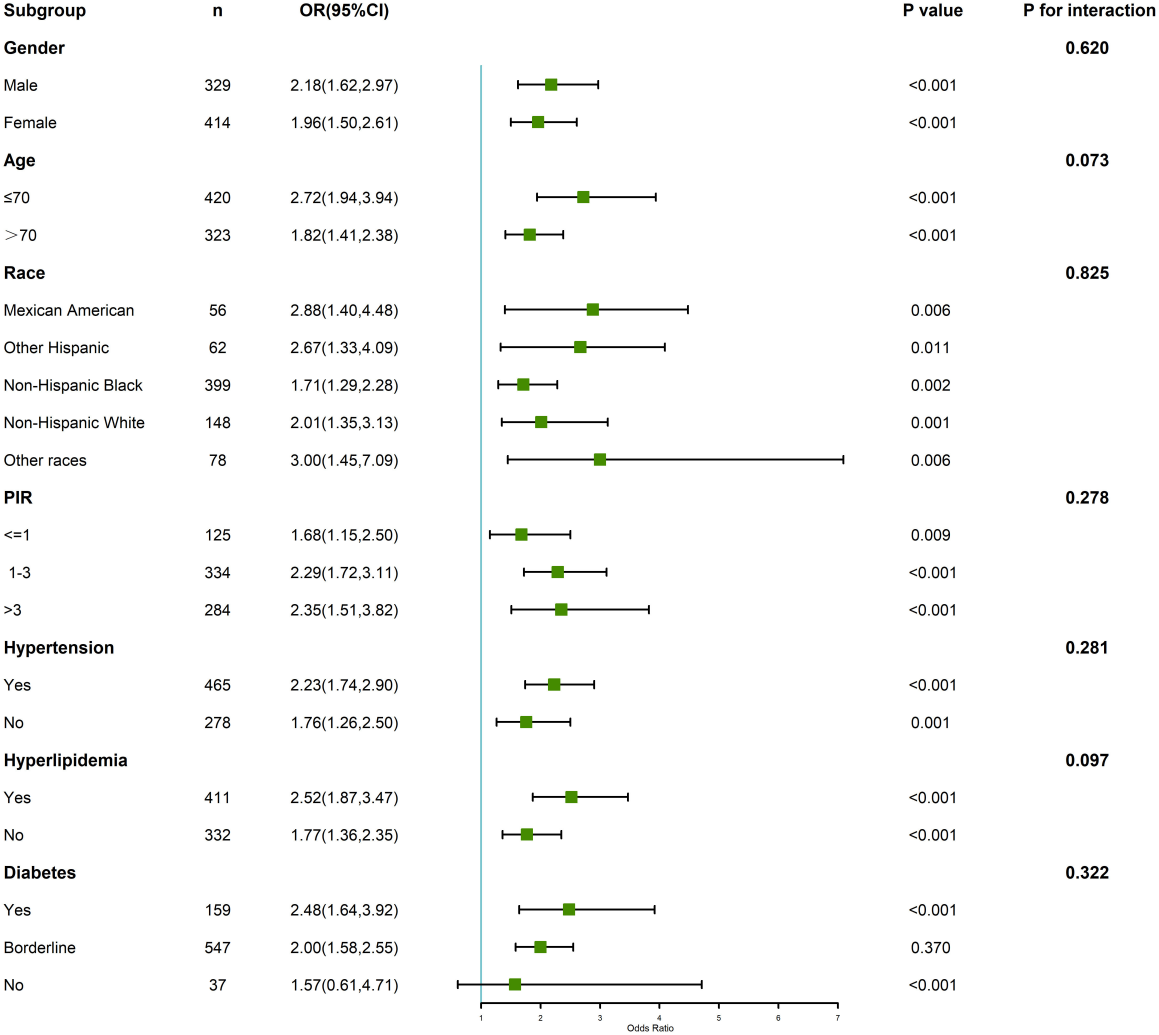
Subgroup and interaction analyses of the association between trihalomethane and cognitive function.

To further explore whether the association between total serum THM concentrations and cognitive impairment varied across population subgroups, we conducted stratified analyses and tested for potential interactions (see Figure 5). Overall, the positive association between high THM exposure and cognitive impairment was more pronounced in several vulnerable groups. Notably, among participants aged ≥70 years, those in the highest quartile of THMs had significantly higher odds of cognitive impairment (OR = 3.72; 95% CI: 2.13–6.47), whereas the association was weaker and not statistically significant in participants < 70 years (OR = 1.71; 95% CI: 0.89–3.28), with a *P*-value for interaction = 0.047. Similar trends were observed by sex: the effect was stronger in females (OR = 3.14; 95% CI: 1.92–5.14) than in males (OR = 1.88; 95% CI: 1.00–3.52), though the interaction was not statistically significant. Participants with hypertension showed a robust association (OR = 3.12; 95% CI: 1.95–4.99), compared to those without hypertension (OR = 1.67; 95% CI: 0.89–3.15), *P* for interaction = 0.038. Likewise, in individuals with diabetes, the association between THM exposure and cognitive impairment was stronger (OR = 3.80; 95% CI: 1.88–7.65) than in non-diabetic individuals (OR = 2.01; 95% CI: 1.14–3.55), with marginal interaction (*P* = 0.062).

No significant interactions were observed by education level or alcohol use; however, the ORs remained elevated in low-education and alcohol-consuming groups. Collectively, these results suggest that older adults and individuals with preexisting metabolic or cardiovascular conditions may be more susceptible to the neurotoxic effects of THM exposure.

## 4 Discussion

This study explores the association between serum trihalomethane (THM) concentrations and cognitive impairment, highlighting significant findings suggesting that higher serum THM levels are associated with an increased risk of cognitive dysfunction. The results align with existing literature that links environmental pollutants, including chlorination by-products, to neurological damage (18). Specifically, we identified that individuals with higher THM levels, particularly bromodichloromethane (BDCM), showed an elevated risk of cognitive impairment, particularly in older adults and those with preexisting conditions like hypertension and diabetes.

The mechanisms through which THMs contribute to cognitive impairment remain incompletely understood. One potential pathway is through oxidative stress, as THMs, particularly BDCM, are known to induce the generation of reactive oxygen species (ROS) in cells, leading to cellular damage in neuronal tissues (19, 20). Previous studies have demonstrated that oxidative stress plays a significant role in neurodegeneration, and chronic exposure to environmental pollutants like THMs could exacerbate this process (21, 22). Furthermore, THMs may also influence neuroinflammatory pathways, which are increasingly recognized as key contributors to cognitive decline (23, 24). Elevated levels of neuroinflammation can disrupt neuronal signaling, impair synaptic plasticity, and lead to neurodegeneration (25). These mechanisms are particularly concerning as they suggest that the neurotoxic effects of THMs could be cumulative and subtle over time, potentially accelerating age-related cognitive decline or contributing to the onset of conditions like Alzheimer's disease and other forms of dementia.

Another potential mechanism is the disruption of the blood-brain barrier (BBB). The BBB plays a crucial role in protecting the brain from harmful substances, but it is vulnerable to damage by environmental toxins, including THMs (26). If THMs or their metabolites can penetrate the BBB, they may directly damage brain tissue, contributing to cognitive dysfunction (27). Additionally, the impact of THMs on neurotransmitter systems, particularly those involved in memory and learning (e.g., acetylcholine), could further exacerbate cognitive impairment, although direct evidence of such effects is limited (28, 29).

From a public health perspective, our findings raise concerns about the widespread exposure to THMs in drinking water, especially given their ubiquitous presence in chlorinated water supplies worldwide. Cognitive impairment and neurodegenerative diseases are growing public health concerns, particularly in aging populations (30). With the increasing prevalence of conditions like dementia, understanding the environmental factors that contribute to cognitive decline is vital (31). Reducing exposure to THMs could, therefore, be a public health priority, particularly in communities that rely on chlorinated drinking water sources. Policy measures to limit THM concentrations in public water supplies and promote the use of alternative disinfection methods, such as ultraviolet treatment or ozonation, could reduce the population's overall exposure to these neurotoxic compounds (32).

However, this study has several limitations. First, the cross-sectional nature of the NHANES data prevents us from establishing causal relationships between THM exposure and cognitive decline. Longitudinal studies are necessary to track the progression of cognitive dysfunction over time in relation to THM exposure (33). Second, while we adjusted for numerous potential confounders, residual confounding remains a possibility, as there may be other unmeasured variables that contribute to both THM exposure and cognitive function. Third, the use of serum THM concentrations as a proxy for long-term exposure may not fully capture the cumulative burden of THM exposure over a lifetime. Future studies should consider using biomarkers of chronic exposure or environmental monitoring data to better assess the long-term effects of THMs. Lastly, while we observed significant associations between THM levels and cognitive impairment, the exact thresholds of exposure that lead to significant neurotoxic effects remain unclear. Further research is needed to identify these thresholds and determine whether any safe levels of exposure exist. Despite adjusting for a broad set of covariates, the possibility of residual confounding remains. Unmeasured factors such as occupational exposures, genetic predispositions, or other environmental pollutants may have influenced the observed associations.

In conclusion, our study provides compelling evidence for the association between higher serum THM concentrations and cognitive impairment, with potential implications for public health policy. The findings suggest that reducing THM exposure, especially in vulnerable populations such as the older person and those with preexisting health conditions, could help mitigate the growing burden of cognitive decline. Nonetheless, more research is needed to clarify the mechanisms behind these associations and to establish causal relationships, as well as to identify effective strategies for reducing THM exposure in the general population.

## Data Availability

The original contributions presented in the study are included in the article/Supplementary material, further inquiries can be directed to the corresponding authors.
