# Supplementary material for: Serum trihalomethanes and cognitive decline: investigating environmental risk factors for neurodegenerative diseases
Source: Front Public Health. 2025 Jun 27;13:1603138. doi: 10.3389/fpubh.2025.1603138 (PMC12245804; doi:10.3389/fpubh.2025.1603138)
Supplement: Supplementary file 1 [file Table_1.docx]

**Supplement Table 1.** Weighted logistic regression analyses between trihalomethanes and cognitive impairment.

|  | **CERAD Test** | | | **Animal Fluency Test** | | | | | | | **Digit Symbol SubstitutionTest** | | | |
| --- | --- | --- | --- | --- | --- | --- | --- | --- | --- | --- | --- | --- | --- | --- |
|  | **Model 1** | **Model 2** | **Model 3** | **Model 1** | **Model 2** | | | **Model 3** | | | **Model 1** | **Model 2** | | **Model 3** |
|  | **OR (95%CI)** | **OR (95%CI)** | **OR (95%CI)** | **OR (95%CI)** | **OR (95%CI)** | | | **OR (95%CI)** | | | **OR (95%CI)** | **OR (95%CI)** | | **OR (95%CI)** |
| Bromoform | 0.99(0.94,1.04) | 0.97(0.92,1.03) | 0.99(0.94,1.04) | 0.99(0.96,1.03) | | 0.99(0.96,1.03) | | | 1.01(0.98,1.04) | | 1.03(0.99,1.06) | | 1.02(0.99,1.05) | 1.05(1.00,1.10) |
| P Value | 0.547 | 0.365 | 0.706 | 0.822 | | 0.729 | | | 0.722 | | 0.170 | | 0.287 | 0.033 |
| Bromodichloromethane | 1.04(0.99,1.09) | 1.04(0.99,1.09) | 1.05(0.99,1.10) | 1.04(0.99,1.08) | | | 1.04(0.99,1.09) | | | 1.05(0.99,1.10) | 1.01(0.97,1.05) | | 1.00(0.96,1.05) | 1.03(0.98,1.08) |
| P Value | 0.068 | 0.123 | 0.076 | 0.096 | | | 0.107 | | | 0.054 | 0.694 | | 0.940 | 0.208 |
| Chloroform | 1.01(0.99,1.02) | 1.01(0.99,1.02) | 1.01(0.99,1.02) | 1.00(0.99,1.01) | | | 1.00(0.99,1.01) | | | 1.00(0.99,1.01) | 1.00(0.99,1.01) | | 1.01(0.99,1.02) | 1.01(0.99,1.02) |
| P Value | 0.261 | 0.190 | 0.197 | 0.806 | | | 0.699 | | | 0.886 | 0.312 | | 0.257 | 0.219 |
| Dibromochloromethane | 1.03(0.97,1.09) | 1.02(0.96,1.09) | 1.04(0.97,1.10) | 1.04(0.99,1.10) | | | 1.04(0.98,1.10) | | | 1.06(0.99,1.12) | 1.03(0.98,1.10) | | 1.03(0.97,1.09) | 1.07(0.99,1.15) |
| P Value | 0.386 | 0.513 | 0.282 | 0.151 | | | 0.156 | | | 0.062 | 0.254 | | 0.414 | 0.057 |
| P for trend | <0.001 | | | <0.001 | | | | | | | <0.001 | | | |

Model 1: no covariates were adjusted.

Model 2: age and sex were adjusted.

Model 3: age, sex, race, education level, marital status, BMI, PIR, smoking status, alcohol status, diabetes status, hypertension status, hyperlipidemia status was adjusted.

95 % CI, 95 % confidence interval.
